# Quality of Life from Return to Work and Sport Activities to Sexual Dysfunction after Surgical Treatment of Pelvic Ring Fractures

**DOI:** 10.3390/healthcare11131930

**Published:** 2023-07-04

**Authors:** Andrea Stefano Monteleone, Pietro Feltri, Jochen Müller, Mauro Natale Molina, Giuseppe Filardo, Christian Candrian

**Affiliations:** 1 Service of Orthopaedics and Traumatology, Department of Surgery, EOC, 6900 Lugano, Switzerland; 2 Faculty of Biomedical Sciences, Università della Svizzera Italiana, 6900 Lugano, Switzerland

**Keywords:** pelvic ring fractures, quality of life, return to work, functional outcomes, sexual function

## Abstract

Background: Pelvic ring fractures are life-threatening injuries that have a severe impact on patients’ lives. The aim of this clinical study was to evaluate the outcome of surgical treatment in terms of Quality of Life (QoL), return to work, functional results and sport activities, and post-operative sexual dysfunction. Methods: A retrospective study with patients retrieved from a Level 1 Trauma Center was performed. Minimum patient follow-up was 12 months: QoL was evaluated with the SF-12 (Short Form Survey) questionnaire, return to work with the Workplace Activity Limitation Survey (WALS), functional outcomes and sport activities with the Harris Hip Score and Tegner activity score, respectively, and sexual function damage with a 0–10 NRS. Results: Seventy-six patients (41 males and 35 females) were enrolled, with a mean age at surgery of 56.4 years (18–89 years). Overall, their quality of life remained significantly affected, with male patients reporting worse WALS outcomes (*p* = 0.036), sexual damage (*p* = 0.001), and SF-12 Bodily Pain (*p* = 0.046) than females. In particular, 70.7% of men and 45.7% of women reported sexual limitations, and only 53.7% returned to their job, with 35.2% losing their job as a consequence of the pelvic ring disruption. Conclusions: An important deterioration in general health state, return to work, and sexual function was documented in patients treated surgically for pelvic trauma, especially in male patients. There are disabling secondary sequels at all levels beyond the mere functional scores, and both patients and clinicians should be aware and have the correct expectations.

## 1. Introduction

Pelvic fractures represent approximately 2–8% of all fractures [1,2], with a yearly incidence of 23 per 100,000 in the general population [3]. Due to their serious consequences, they are a major concern for orthopaedic and traumatology surgeons. The majority of pelvic ring disruption is due to high-energy trauma such as car and motorcycle accidents (80.5%) or falls from great heights (10.5%), while low-energy trauma is less common (9%) [4]. Thus, pelvic fractures often represent life-threatening injuries whose outcomes are variable and uncertain, as they depend on the dynamics of the trauma, the localization and extent of the injury, associated lesions, and appropriate treatment. Numerous attempts have been made to improve the surgical treatment of pelvic fractures; in this light, the classifications of Tile and Young-Burgess [5,6] have provided a solid basis for their management, leading to the development of several surgical approaches based on the radiological type of fracture.

Due to the not-common occurrence of these severe fractures and the limited literature on large series of patients, little is known about the Quality of Life (QoL) and the return to work and sports activities after pelvic fractures [7,8,9,10,11]. At the time of trauma, neurological damage at the lumbar and sacral plexuses might occur; thus, results are often not optimal, with some reports suggesting, on the one hand, the occurrence of sexual dysfunction and, on the other hand, difficulties in fully returning to work [12,13,14,15,16]. Therefore, pelvic fractures may play an important role not only for the healthcare system, but they can be so severe in disrupting patient life as to affect QoL and socioeconomic status as well. Even though these are key outcomes to understand the impact of these fractures, nowadays very few publications assessing QoL, return to work or sport activity, and sexual dysfunction after pelvic ring fractures have been published. This hinders the possibility of understanding patient and fracture-related prognostic factors, presenting reliable expectations to patients undergoing surgery, and possibly identifying useful aspects to optimize the management of patients affected by pelvic ring disruptions.

The aim of this study was to document the outcomes of pelvic fractures in terms of QoL, return to work, and sport activities, as well as post-operative sexual dysfunction, through the evaluation of a large series of patients treated for pelvic fractures in a Level 1 Trauma Center.

## 2. Materials and Methods

### 2.1. Patient Enrolment

This study was conducted at a Level 1 Trauma Center after the approval of the local Ethical Committee (2021-00231/CE 3815). After informed consent, the patients surgically treated for a pelvic ring fracture from January 2010 to March 2020 were evaluated. Patients unable to provide informed consent, with only acetabular fractures, or with pathological fractures (i.e., metastatic, osteoporotic) were excluded.

Data from patient medical histories were collected from the hospital registers, including patients’ characteristics, diagnosis, surgery, and post-operative complications, and an interview was conducted at a minimum of 12 months follow-up after surgery. To minimize the risk of bias, all interviews were conducted by the same physician, with the supervision of a second author consulted in case of doubt. A STROBE checklist was used in order to prepare the scientific article.

### 2.2. Patient Evaluation

Patient-related QoL and health state were assessed using the American 12-Item Short-Form Health Survey (SF-12) [17]. The 2009 U.S. general population norm was set, where the Physical Component Scores (PCS) and the Mental Component Scores (MCS) were standardized with a mean of 49.2 ± 15.1 and 53.8 ± 13.1 points, respectively [18]. Return to work was assessed using the Workplace Activity Limitation Survey (WALS) proposed by Gignac [19]. The differentiation of the types of work was based on the Canadian Classification and Dictionary of Occupations (CCDO) [20]. Functional outcomes were assessed through the Harris Hip Score (HHS) and the Tegner activity score [21,22]. The negative impact of damages to the sexual apparatus was evaluated on a NRS scale by asking the patients to rate from 0 to 10 the worsening in their sexual life due to the pelvic trauma (10 being the worst outcome). Post-operative major complications checked in the medical records were damages to the peri-pelvic organs, vascular system or thrombosis, and nervous system; death related to the trauma; and re-intervention associated with the initial injury. All questions were based only on factors related to the pelvic ring fractures.

### 2.3. Statistical Analysis

The statistical analysis was performed using SPSS v.19.0 (IBM Corp., Armonk, NY, USA) [23].

All continuous data were expressed in terms of mean ± SD, median, and range; categorical variables were expressed as proportions or percentages. The Shapiro–Wilk test was performed to test the normality of continuous variables. The Levene test was used to assess the homoscedasticity of the data. The ANOVA test followed by the Scheffè post hoc pairwise comparison was used to assess the differences among groups of continuous, normally distributed, and homoscedastic data; the Kruskal–Wallis test followed by the Mann–Whitney test with the Bonferroni correction for multiple comparisons was used otherwise. The Monte Carlo method was used to evaluate the non-parametric tests due to the small size of the sub-groups. The Pearson’s chi-squared exact test for small samples was performed to investigate relationships between grouping variables. The Spearman Rank Correlation was used to assess correlations between numerical scores and continuous data.

For all tests, *p* < 0.05 was considered significant.

## 3. Results

### 3.1. Patients’ Characteristics

Out of 90 patients (52 men and 38 women) asked to participate in the study, 76 (84.4%) agreed to be enrolled, while 13 were not reachable and one refused to participate in the interview. The mean age was 56.4 ± 20.7 (range: 18–89 years old) at the time of surgery. Patients were assessed at a mean of 62.2 ± 35.9 months (12–132 months) after trauma. Patients’ demographics, mechanism of injury, post-operative complications, and time between injury and follow-up are detailed in Table 1.

Among the causes of injury (Table 1), the predominant was falling, as it occurred in 45 patients (59.2%), while 24 patients (31.6%) were brought to the emergency room because of road traffic incidents. Six patients (7.9%) had a job injury reported in the medical records as “injury due to crushing from material”, and one patient (1.3%) had a pelvic ring fracture caused by a skiing injury. Among the 45 cases of falls, 14 patients (18.4%) fell from a great height (above 3 m), 14 patients (18.4%) fell from a low height (1–3 m), and 17 patients (22.4%) fell from a standing height. Post-operative complications are reported in detail in Table 1. The causes of trauma were statistically different between males and females (*p* = 0.002), with males needing emergency treatment due to more high-energy traumas (road traffic incidents, falls greater than 3 m, work injuries) than low ones (standing height falls) compared to females. The patients in the current study were treated either with 1 plate, 1 ASNIS, 2 ASNIS, or a combination of plate and ASNIS screws based on the type of injury (Table 1).

Data regarding the direction of force causing the pelvic injury are shown in Table 2, where they have been stratified according to the Young and Burgess classification [6] and according to the Tile classification [5].

Nineteen patients (25.0%) had at least one more surgery related to the original trauma. The main cause (10 patients—13.2%) was the removal of the fixation device, due to pain or the desire to become pregnant. Other causes of reintervention were the mobilization of fixation devices (7.9%) and post-traumatic osteoarthritis that led to femoral head arthroplasty (5.3%). A bilateral femoral head arthroplasty due to post-traumatic osteoarthritis was performed during the same surgery on a 61-year-old patient. Forty-two of the seventy-six patients included in the study presented concomitant injuries. The majority (71.4%) sustained vertebral fractures, but one quarter of them had to undergo surgery. The second most common injuries were thorax traumas like rib fractures, lung contusions, and pneumothorax that did not require surgeries. The majority of the patients having surgery for associated lesions had lower/upper limb fractures. It was found that women had statistically fewer concomitant lesions than men (*p* = 0.02). Further statistical analysis was not performed because of the high heterogeneity of the different types of concomitant injuries.

### 3.2. Quality of Life

Patients’ SF-12 PCS and MCS were 43.0 ± 9.1 and 50.0 ± 9.4 points, respectively. The mean and median scores for all SF-12 subscales are summarized in Table 3. When analyzing each SF-12 domain, the highest score is found except the Social Functioning and the lowest score in Role Physical. In general, all the retrieved SF-12 values but the Social Functioning were below the US-based norm population [18].

Results stratification based on the Tile or the Young and Burgess classifications found no statistical differences among the subgroups neither in the PCS nor the MCS (n.s.); the lowest PCS results were identified in the lateral compression 2 (LC2) (38.2 ± 9.5). The analysis of the results by gender showed a statistical difference (*p* = 0.046) in the Bodily Pain scale between males and females, with men having a worse outcome. Age was directly correlated with the SF-12 Bodily Pain Scale (*p* = 0.002, Rho = 0.357).

### 3.3. Return to Work

Of the 76 enrolled patients, 22 (28.9%) were retired at the time of injury; therefore, the WALS questionnaire was not administered to those patients. The WALS questionnaire of the working population presented an overall score of 27.1 ± 12.1. Out of the 54 working-active patients, 29 (53.7%) fully returned to work in the same position and duties as before the trauma. A change at work was observed in 25 patients (46.3%): among these, 19 (35.2%) lost their job, and six (11.1%) were requalified with a new job that did not require the same physical effort as before.

Stratified according to gender, males reported the worst results (*p* = 0.036) with a score of 29.9 ± 12.4 compared to 22.4 ± 10.2 in females (Table 4). Stratified according to gender, in the very heavy labor category, 18 were men and 3 were women. Among them, 14 men and 3 women lost their jobs. No woman had a heavy job. In the moderate category, 6 were men and 13 were women. Among them, 66.7% of men and 23.1% of women lost their jobs. In the light labor category, 9 were men and 5 were women, and only one man lost his job.

**Figure 1:**
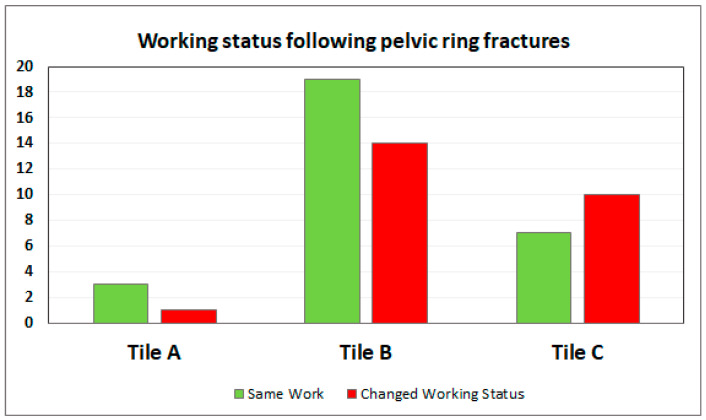
Working status following the pelvic ring fracture surgery. An increase in job loss was observed as the severity of the fractures increased. In the vertical axis the number of patients is represented.

Of the 25 patients who were fired or requalified for a new job, 17 (68.0%) had a heavy or very heavy job before the trauma (81.0% of those with a heavy or very heavy job lost their job). No statistical difference was found among Tile subgroups or among the Young and Burgess classifications (Table 5). Even though their difference did not reach statistical significance, an increase in job loss was observed as the severity of the fractures increased: job loss reached 25.0% in the A-types, 40.6% in the B-types, and 61.1% in the C-types (Figure 1). Fractures due to Vertical Shear (VS) had the highest percentage of job loss (85.7%), while LC1 had the lowest percentage (25.0%). It might be crucial to state that the type of work and the type of trauma (low energy vs. high energy) were not statistically correlated with any of the outcomes (*p* > 0.05) and that the causes of trauma did not significantly influence the job loss (*p* > 0.05).

Table 4 shows the outcomes of surgically treated pelvic ring fractures. Results were stratified according to the Tile classification and according to gender. Statistically significant differences have been found in the WALS and in sexual functional damage between men and women.

Table 5 shows the outcomes of surgically treated pelvic ring fractures. Results have been stratified according to the Young and Burgess classifications. A statistically significant difference has been found in sexual functional damage.

### 3.4. Functional Outcomes and Sport Activities

The mean values of the functional outcomes assessed through the HHS and Tegner activity scores were 72.9 ± 18.8 and 2.6 ± 1.8, respectively. No statistical difference was found among Tile subgroups (Table 4) nor among the Young and Burgess classification (Table 5); the lowest score values were reported in the C2 group for HHS (67.1 ± 33.8) and in the B2 group for Tegner (2.2 ± 1.5). Age was identified to be inversely correlated with HHS (*p* = 0.003, Rho = 0.333) and Tegner activity score (*p* < 0.001, Rho = 0.504). To evaluate the influence of the duration of follow-up on the outcomes, the cohort was divided into 5 groups (every 24 months of follow-up). Twelve patients had a follow-up between 2 and 24 months, 25 between 25 and 48 months, 12 between 49 and 72 months, 8 between 73 and 96 months, and 19 between 97 and 121 months. No statistical difference regarding the outcomes was found among different patient groups based on the follow-up time.

### 3.5. Sexual Functional Damage

Twenty-nine males (70.7%) and sixteen females (45.7%) reported a change in their sexual habits after the pelvic fracture. The mean change after the injury in the male population was 4.2 ± 3.6, while in the female population it was 1.4 ± 2.3. Gender significantly influenced the results (*p* = 0.001), with males reporting the worst outcomes. No correlation was found between the time of follow-up and sexual dysfunction. All of the patients who experienced sexual dysfunction were asked if they had organic damage, and the medical records of each patient were checked. Out of the 45 patients who reported impaired sexual function after the trauma, 32 stated that they had no organic damage; 10 patients suffered from nerve lesions unrelated to sexual function; 2 suffered from chronic pain refractory to any type of therapy; and 1 was paraplegic due to the trauma. The patients reported no coping mechanisms. A statistical difference in sexual outcomes was seen when the patients were stratified according to the Young and Burgess classification (*p* = 0.035), as presented in Table 5. The majority of males among those with sexual damage (*n* = 20/29; 68.9%) had an overall score equal to or higher than five points (out of 10), reporting a very important change in their sexual habits (i.e., sexual dysfunction, pain during intercourse). On the other hand, in the female population, only a few patients among those who reported a change in their sexual function (*n* = 3/16; 18.8%) had a score equal to or higher than five, with the majority reporting a slight change (i.e., pain during intercourse). Age was identified as being inversely correlated with sexual damage score (*p* < 0.001, Rho = 0.409).

### 3.6. Low vs. High Energy Traumas

When analyzing the general population, a statistical difference was found in the Tegner score (*p* = 0.006) and in the sexual damage score (*p* = 0.001), with patients presenting to the emergency department because of high energy traumas resulting in the worst sexual damage scores but a better Tegner value. Injuries were divided as occurring in people < or >65 years old: 29 (38.2%) were >65 years old. Of them, 17 patients sustained a low energy trauma, while the other 12 sustained a high energy trauma. The comparison of these groups showed no statistical difference in all the outcomes in addition to the Tegner score, which was higher in those with high energy trauma (*p* = 0.012). Geriatric patients who did not undergo surgery were excluded from the study.

## 4. Discussion

The main finding of this study is that pelvic fractures lead to a significant worsening of patients’ quality of life, as documented by scores exploring different spheres of patients’ lives. In fact, this goes beyond a mere reduction in functional scores, leading to a high rate of job loss and a significant probability of sexual dysfunction, especially for male patients.

Patients QoL is a key outcome to assess their results after surgical treatment of pelvic ring fractures. For the purpose of this study, a validated questionnaire was chosen to define QoL; the evaluation through the SF-12 showed low QoL in both physical and mental scales compared to the norm population, as well as in all the subscales but Social Functioning [18]. Thus, while patients seem to cope at a social function level, they suffer from a QoL worsening, reaching the most impactful consequences for bodily pain, with significant effects both at a physical and emotional level. Therefore, psychological and social functions should be taken into account to gain an overall picture of the patient’s health perspective. These results underline the complex effects on patient QoL, building upon previous reports suggesting an overall impact on the pelvic fracture patient’s health. Borg et al. reported in 2010 [10] that patients with pelvic ring fractures scored worse than the general population on the SF-36 score at a two-year follow-up. The administration of the SF-questionnaire for these patients was explored by Oliver et al. [24], with 35 patients having a 14% lower PCS and a 5.5% lower MCS than the normative population. Van den Bosch et al. [25] reported lowered SF-36 results at almost 3-year follow-up in a series of 37 patients. These authors reported the worst results in the PCS, especially in the Role Physical, despite good radiological results. The severity of the radiological presentation seemed important in determining the patient’s QoL, although this correlation is still under debate and most studies do not differentiate between the Tile sub-groups when evaluating the QoL of the treated patients. As a matter of fact, Rommens and Hessmann [26] underlined that patients with anterior and posterior instability, as in Tile C-type fractures, are characterized by a higher risk of unfavorable functional outcomes than B-type fractures. Even without reaching statistically significant results, the findings of the current article are in line with the few data points present in the literature so far, retrieving the lowest scores in the C-type fractures. This underlines the importance of further exploring the effects of pelvic fractures by documenting a large series of patients to understand lesion and patient factors that might influence the final outcome. In addition to the overall impact on patient QoL, other aspects should be explored to investigate patient outcomes and their impact on their lives.

To understand the real effects in terms of QoL and the possibility of going back to their previous everyday lives, it is crucial to consider the return to work and the level of return to previous work-related activities. This study identified a significant impact in terms of return to work, with only one in every two patients going back to his previous position. One in every three patients even lost their job, with a higher job loss in patients with Tile B-types (40.6%) and C-types (61.1%) and with Young and Burgess vs. fractures (85.7%). These results expand on what was suggested by previous literature findings [13,14,15,27,28,29]. Papasotiriou et al. [14] examined the return to work of 77 patients and identified the highest percentage of job loss in C-type fractures (66.7%). Not only the type of fracture but also associated injuries and job type have been identified as prognostic factors for returning to work after pelvic ring injuries [15,27,30]. In line with these findings, this study found that 81.0% of the patients who had a heavy or very heavy job did not return to their previous work.

Regardless of the possibility of resumed work activities, a severe impact was found in a more intimate sphere. The evaluation of sexual function showed persistent damage in most patients. However, the impact was different for different types of patients. In particular, the analysis stratified according to gender showed that females were less affected. On the other hand, 70% of men were affected and the majority with significant impairment. This data documented the sexual sphere as a whole, evaluated from the patient’s perspective, offering an important addition to what was previously investigated. Previous studies looking specifically at a single outcome measure when assessing the sexual damage, especially organic damages, underlined that men were more likely to suffer from erectile dysfunction [31,32,33,34], while women suffered from dyspareunia [35]. Harvey-Kelly et al. [11] published a retrospective study in 2014 where 80 surgically treated pelvic patients filled in multidimensional questionnaires about sexual life twice, once regarding their state at follow-up and once in retrospect regarding their state before the injury. In their study, sexual dysfunction was identified in both genders, with males having a significantly higher rate than females (52.1% vs. 43.8%) [11]. In this study, an even higher percentage of sexual functional damages were documented when asking men to report on their sexual sphere.

Gender is an important factor, not only in terms of sexual function-related results. The mechanism of trauma was found to be statistically different between males and females, with men reporting more high-energy traumas, which could explain the lower outcome in terms of sexual function and, more generally, in the SF-12 Bodily Pain scale and the ability to return to work. Gender stratification of patients self-reported QoL has been scarcely investigated in the literature so far, with controversial findings. Hernefalk et al. in 2019 [36] prospectively studied the long-term functional outcome of surgically treated pelvic ring B- or C-type disruptions. In contrast to the current study, they reported worse functional outcomes in females at a 5-year follow-up, both in the generic (SF-36) as well as in other condition-specific outcome measures. However, the difference between male and female mechanisms of injury was not evaluated in that series [36]. The lack of baseline gender analysis on the mechanism of injury hinders the possibility of interpreting if those findings were driven by a higher number of high-energy traumas or by specific sex-related characteristics of the female group. In this light, gender stratification should be considered in future studies, as this aspect can significantly influence the results, as documented in the current series on different spheres of patients’ QoL.

This study investigation takes gender-related results into account while documenting different important aspects of the results of a large series of patients treated surgically for pelvic fractures. Still, this study presents some limitations. The retrospective nature of the study did not allow for comparison of the follow-up scores with values before the trauma. However, QoL is commonly documented with reference to norm values, which were used as references for the purpose of this study. This limitation was overcome by the other major findings of this study. In this regard, both sexual damage and return-to-work scores were related to the changes with respect to patients’ usual activities before trauma. Being that these questions were not associated with a specific moment in time but with everyday patients’ experiences, a recollection bias was minimized. We referred to patients all presenting a minimum follow-up time to make sure that the documented patient outcomes were stabilized after surgical treatment. Another limitation was the relatively small number of some subcategories when the data were stratified according to the Tile [5] and the Young and Burgess classifications [6]. However, a subanalysis based on specific subcategories was not the purpose of this study, which, on the other hand, presented a large series treated and documented in a Level 1 Trauma Center and offered important insights for this field. All patients were evaluated from both an objective and subjective perspective, combining medical records and direct patient assessment with validated questionnaires. Last but not least, the gender stratification of both the mechanisms of injury and the different results was able to provide significant results for this analysis.

Pelvic trauma fractures have an overall impact on different spheres of patient QoL, ranging from symptoms to function and with consequences for both work and sexual activities. This should be considered by patients and physicians to have the correct expectations in terms of recovery after trauma. Finally, understanding the impact of the lesion and patient types on the final outcome could help develop more targeted treatments to optimize the management of patients with pelvic ring fractures.

## 5. Conclusions

An important deterioration in general health state, return to work, and sexual function was documented in patients treated surgically for pelvic trauma, especially in male patients. Most men had persistent and serious sexual dysfunction and could not go back to heavy or very heavy jobs. There are disabling secondary sequels at all levels beyond the mere functional scores, and both patients and clinicians should be aware and have the correct expectations. Further understanding the nature and types of patients affected by permanent dysfunctions may help optimize the complex management of pelvic ring fractures.

## Figures and Tables

**Table 1:** Baseline characteristics of the study group.

**Gender**	
Male, N; %	41 (53.9%)
Female, N; %	35 (46.1%)
Age (y), mean; SD	56.4 ± 20.7
Follow-up (m), mean; SD	62.2 ± 35.9
**Mechanism of injury**	**N (%)**
Road Traffic Incidents	24 (31.6%)
MVA	4 (5.3%)
MCA	15 (19.7%)
Pedestrian	5 (6.6%)
Fall	45 (59.2%)
Standing height	17 (22.4%)
Low height (1–3 m)	14 (18.4%)
High height (>3 m)	14 (18.4%)
Work injury	6 (7.9%)
Ski	1 (1.3%)
**Post-Op complications**	**N (%)**
Neurological	16 (21.1%)
Sphincter	2 (2.6%)
Peri-pelvic organs	4 (5.3%)
**Type of surgery**	**N (%)**
One ASNIS screw	2 (2.6%)
Two ASNIS screws	34 (44.7%)
Plate	30 (39.5%)
Plate with ASNIS screws	10 (13.2%)
**Re-Op**	**N (%)**
Removal of fixation devices	10 (13.2%)
Mobilization of fixation devices	6 (7.9%)
PTA to FHA	4 (5.3%)

N—number of cases; y—years; SD—standard deviation; m—months; MVA—motor vehicle accident; MCA—motorcycle accident; Op—operation; PTA—post-traumatic arthritis; FHA—femoral head arthroplasty.

**Table 2:** Number of pelvic ring fractures based on Tile, Young, and Burgess classifications.

**Tile**	**N**	**%**
A (A1, A2, A3)	6 (0, 5, 1)	7.9%
B (B1, B2, B3)	46 (14, 19, 13)	60.5%
C (C1, C2, C3)	24 (12, 3, 9)	31.6%
**Young and Burgess**	**N**	**%**
APC (APC1, APC2, APC3)	30 (0, 6, 24)	31.3%
LC (LC1, LC2, LC3)	45 (24, 6, 15)	46.9%
VS	11	11.5%
CM	10	10.4%

N—number of cases; APC—anteroposterior compression type; LC—lateral compression type; VS—vertical shear type; CM—combined mechanism of injury type.

**Table 3:** SF-12 results in 76 surgically treated patients following pelvic ring fractures compared with norm-based values.

**Scales**	**Mean (SD)**	**Median (Range)**	**US General Population**
SF-12 (Physical Component Score)	43.0 (9.1)	42.9 (21.9–59.4)	49.2 (15.1)
SF-12 (Mental Component Score)	50.0 (9.4)	51.4 (24.9–69.1)	53.8 (13.1)
**Subscales**	**Mean (SD)**	**Median (range)**	**US general population**
Physical Functioning	45.8 (10.2)	49.2 (25.6–57.1)	50.7 (14.5)
Role Physical	41.2 (9.9)	40.5 (23.6–57.5)	49.5 (14.7)
Bodily Pain	42.5 (12.9)	48.7 (21.7–57.7	50.7 (16.3)
General Health	45.0 (9.0)	47.8 (23.9–57.7)	50.1 (16.9)
Vitality	49.7 (9.4)	49.1 (29.4–68.7)	53.7 (15.4)
Social Functioning	52.5 (7.5)	56.9 (30.2–56.9)	51.4 (13.9)
Role Emotional	43.9 (11.9)	45.9 (19.9–56. 3)	51.4 (13.9)
Mental Health	48.4 (9.6)	47.0 (24.1–64.2)	54.3 (13.3)

SD—standard deviation; US—United States; SF—American 12-Item Short-Form Health Survey.

**Table 4:** Outcomes of surgically treated pelvic ring fractures (Tile).

Questionnaires	N	Mean (SD)	Median (Range)	*p*-Value
**WALS** (12–48, 48 worst score)	54	27.1 (12.1)	26.0 (12–48)	
Tile–A	4	23.8 (18.3)	20.5 (12–48)	0.720
Tile–B	32	26.6 (11.2)	24.0 (12–48)	
Tile–C	18	28.7 (12.8)	34.0 (12–48)	
Male	34	29.9 (12.4)	30.5 (12–48)	**0.036**
Female	20	22.4 (10.2)	20.5 (12–48)	
**HHS** (0–10, 0 worst score)	76	72.9 (18.8)	75.7 (8.4–100)	
Tile–A	6	71.7 (19.2)	63.7 (53.9–96)	0.988
Tile–B	46	72.9 (19.2)	73.6 (8.4–100)	
Tile–C	24	73.1 (18.6)	79.0 (23.2–96)	
Male	41	73.3 (22.0)	81.5 (8.4–96)	0.332
Female	35	72.4 (14.4)	71.3 (49.0–100)	
**Tegner activity score** (0–10, 0 worst score)	76	2.6 (1.8)	2.0 (1.0–7.0)	
Tile–A	6	2.7 (1.5)	3.0 (1.0–5.0)	0.242
Tile–B	46	2.3 (1.7)	2.0 (1.0–7.0)	
Tile–C	24	3.1 (1.9)	3.0 (1.0–7.0)	
Male	41	2.9 (1.9)	3.0 (1.0–7.0)	0.100
Female	35	2.2 (1.6)	2.0 (1.0–7.0)	
**Sexual functional damage** (0–10, 0 worst score)	76	2.9 (3.3)	1.5 (0–10)	
Tile–A	6	2.2 (3.1)	0.5 (0–7)	0.200
Tile–B	46	2.5 (3.2)	1.0 (0–10)	
Tile–C	24	3.9 (3.6)	2.5 (0–10)	
Male	41	4.2 (3.6)	4.0 (0–10)	**0.001**
Female	35	1.4 (2.3)	0.0 (0–9)	

N—number of cases; SD—standard deviation; WALS—Workplace Activity Limitation Survey; HHS—Harris Hip Score.

**Table 5:** Outcomes of surgically treated pelvic ring fractures (Young and Burgess).

Questionnaires	N	Mean (SD)	Median (Range)	*p*-Value
**HHS** (0–10, 0 worst score)	76	72.9 (18.8)	75.7 (8.4–100)	
APC	21	74.5 (19.4)	82 (37.5–96.0)	0.904
LC	36	71.8 (19.6)	72.3 (8.4–100.0)	
VS	9	72.8 (12.0)	79 (53.6–82.9)	
CM	10	73.6 (21.8)	75.3 (23.2–96.0)	
**Tegner activity score** (0–10, 0 worst score)	76	2.6 (1.8)	2 (1.0–7.0)	
APC	21	3.2 (2.2)	3 (1.0–7.0)	0.051
LC	36	2.1 (1.5)	2 (1.0–7.0)	
VS	9	3.0 (0.9)	3 (2.0–5.0)	
CM	10	2.7 (2.2)	1.5 (1.0–7.0)	
**Sexual functional damage** (0–10, 0 worst score)	76	2.9 (3.3)	1.5 (0–10)	
APC	21	2.3 (3.3)	0 (0–10)	**0.035**
LC	36	2.3 (3.0)	1 (0–10)	
VS	9	4.7 (3.6)	7 (0–9)	
CM	10	4.8 (3.6)	5 (0–10)	

N—number of cases; SD—standard deviation; HHS—Harris Hip Score; APC—anteroposterior compression type; LC—lateral compression type; VS—vertical shear type; CM—combined mechanism of injury type.

## Data Availability

Data supporting results were obtained and were inserted in a database of the EOC Lugano Hospital after the study’s approval from the local Ethical Committee Software. The software is approved by the Ethical Committee and is named 4DBase.
